# Peripheral Antinociception Induced by Aripiprazole Is Mediated by the Opioid System

**DOI:** 10.1155/2017/8109205

**Published:** 2017-07-03

**Authors:** Renata Cristina Mendes Ferreira, Ana Flávia Almeida-Santos, Igor Dimitri Gama Duarte, Daniele C. Aguiar, Fabricio A. Moreira, Thiago Roberto Lima Romero

**Affiliations:** Departamento de Farmacologia, Instituto de Ciências Biológicas, Universidade Federal de Minas Gerais, Belo Horizonte, MG, Brazil

## Abstract

**Background:**

Aripiprazole is an antipsychotic drug used to treat schizophrenia and related disorders. Our previous study showed that this compound also induces antinociceptive effects. The present study aimed to assess the participation of the opioid system in this effect.

**Methods:**

Male Swiss mice were submitted to paw pressure test and hyperalgesia was induced by intraplantar injection of prostaglandin E_2_ (PGE_2_, 2 *μ*g). Aripiprazole was injected 10 min before the measurement. Naloxone, clocinnamox, naltrindole, nor-binaltorphimine, and bestatin were given 30 min before aripiprazole. Nociceptive thresholds were measured in the 3rd hour after PGE_2_ injection.

**Results:**

Aripiprazole (100 *μ*g/paw) injected locally into the right hind paw induced an antinociceptive effect that was blocked by naloxone (50 *μ*g/paw), a nonselective opioid receptor antagonist. The role of *μ*-, *δ*-, and *κ*-opioid receptors was investigated using the selective antagonists, clocinnamox (40 *μ*g/paw), naltrindole (15, 30, and 60 *μ*g/paw), and nor-binaltorphimine (200 *μ*g/paw), respectively. The data indicated that only the *δ*-opioid receptor antagonist inhibited the peripheral antinociception induced by aripiprazole. Bestatin (400 *μ*g), an aminopeptidase-N inhibitor, significantly enhanced low-dose (25 *μ*g/paw) aripiprazole-induced peripheral antinociception.

**Conclusion:**

The results suggest the participation of the opioid system via *δ*-opioid receptor in the peripheral antinociceptive effect induced by aripiprazole.

## 1. Introduction

Aripiprazole is an antipsychotic drug with a complex pharmacology. Its main mechanism of action consists in the partial agonism at dopamine D_2_ receptor [1]. In investigating the behavioral pharmacology of aripiprazole in experimental animals, we showed that this compound also decreases the PGE_2_-induced hyperalgesia in a dose-dependent manner in the mechanical paw withdrawal test [1]. In addition, systemic administration of this compound reduced the licking time in the second phase of the formalin test and in the latency time of tail flick test [2].

Recently, it was demonstrated that neurons expressing the dopaminergic receptors were immunopositive for the endogenous opioid met-enkephalin [3]. Met-enkephalin is produced after the cleavage of precursor peptide proenkephalin (PENK) protein. Other endogenous opioids include endorphin, formed after the cleavage of the precursor proopiomelanocortin (POMC) protein, and dynorphins derived from cleavage of prodynorphin. Endogenous opioids may bind preferentially to one of the three opioid receptors. Enkephalin has higher affinity for *μ* opioid receptors, whereas endorphin binds to *μ* and *δ*-opioid receptors and prodynorphin exhibits higher affinity for opioid *κ*-receptors [4]. Opioid receptors are metabotropic receptor coupled to Gi protein. Once activated by agonists, such as morphine or endogenous opioid peptides, they lead to the inhibition of adenylate cyclase and reduction of cAMP synthesis [5]. They also impair extracellular calcium influx and inhibit cell depolarization [6–8].

The opioid system is widely distributed in the peripheral and central nervous system (CNS) [9]. They have been implicated in peripheral antinociception induced by nonopioidergic compounds, including nonsteroidal anti-inflammatory drugs [10] and *α*2-adrenergic agonists [11–13]. Therefore, the peripheral study could be a tool to minimize side effects and to facilitate drugs administration. Thus, considering this context, the aim of the present study was to test the hypothesis that opioid receptors mediated the antinociceptive effect of aripiprazole.

## 2. Materials and Methods

### 2.1. Animals

The present study was approved by the Committee for Ethics in Animal Experimentation (CEUA) under the protocol number 109/2011. Every effort was made to minimize any suffering of animals. All experiments were performed on 30–35 g (aged: 3 months) male Swiss mice and were kept in a cage of size 30 × 35 cm, with 10 animals in each cage in a room maintained at 25 ± 1°C with a 12 h light/dark cycle (6:00 a.m.–6:00 p.m.). Each animal was used only once. Food and water were available ad libitum.

### 2.2. Drugs

Aripiprazole was injected subcutaneously into the plantar surface of the right hind paw (25 and 100 *μ*g). The substance was provided by Bristol-Myers Squibb (Syracuse, NY, USA) and Otsuka Pharmaceuticals (Naruto, Tokushima, Japan). It was dissolved in physiological saline containing 5% tween 80; prostaglandin E_2_ (PGE_2_; hyperalgesic agent; Sigma®) was diluted in ethanol [14]. All other drugs used were diluted in saline, naloxone (nonselective antagonist at opioid receptors, Sigma), bestatin (an aminopeptidase-N inhibitor, Tocris®), clocinnamox (selective *μ*-opioid receptor antagonist, Sigma), nor-Binaltorphimine dihydrochloride (nor-BNI, selective *κ*-opioid receptor antagonist, Sigma), and naltrindole (selective *δ*-opioid receptor antagonist, Tocris).

### 2.3. Measurement of Hyperalgesia

Hyperalgesia was induced by subcutaneous injection of PGE2 (2 *μ*g) into the plantar surface of the right hind paw and it was measured according to the paw pressure test described by Randall and Selitto [14] and modified by Kawabata and coworkers [15]. An analgesiometer (Ugo-Basile, Italy) with a cone-shaped paw-presser with a rounded tip was used to apply a linearly increasing force to the right hind paw of the mice. The weight in grams required to elicit a nociceptive response, the paw withdrawal threshold, was determined as the nociceptive threshold. A cutoff value of 160 g was used to reduce the possibility of damaging the paw. The nociceptive threshold was measured in the right paw and determined by the average of three consecutive trials recorded before (zero time) and 3 hours after PGE2 injection (peak of action). The results were calculated by the difference between these two averages (Δ of nociceptive threshold) and expressed in grams. To reduce stress, the mice were habituated to the apparatus 1 day prior to the experiments.

### 2.4. Experimental Protocols

In all experiments the baseline threshold of each animal was first determined before the injection of any substance. PGE_2_ (2 *μ*g) was given and the nociceptive responses were measured after 180 minutes. To evaluate the antinociceptive peripheral effects of aripiprazole, this compound (25 or 100 *μ*g) was given into the right paw 170 min after PGE_2_ injection. To test if its effects would be inhibited by naloxone (50 *μ*g), naltrindole (15, 30, and 60 *μ*g), clocinnamox (40 *μ*g), or nor-BNI (200 *μ*g), these antagonists were given 140 min and aripiprazole (100 *μ*g) 170 min after PGE_2_ injection. To investigate the effects of an aminopeptidase-N inhibitor, bestatin (400 *μ*g) was administered 140 min prior to PGE_2_. The protocols concerning dose and time of administration of each drug used in this study were obtained through pilot experiments and literature data [16, 17].

### 2.5. Statistical Analysis

The data were analysed with the GraphPad Prism 5 Software®. Drug treatments were compared by one-way analysis of variance (ANOVA). Post hoc analyses were performed with the Bonferroni test. All data are expressed as the mean and SEM statistical difference was set as *p* < 0.05.

## 3. Results

**Figure 1:**
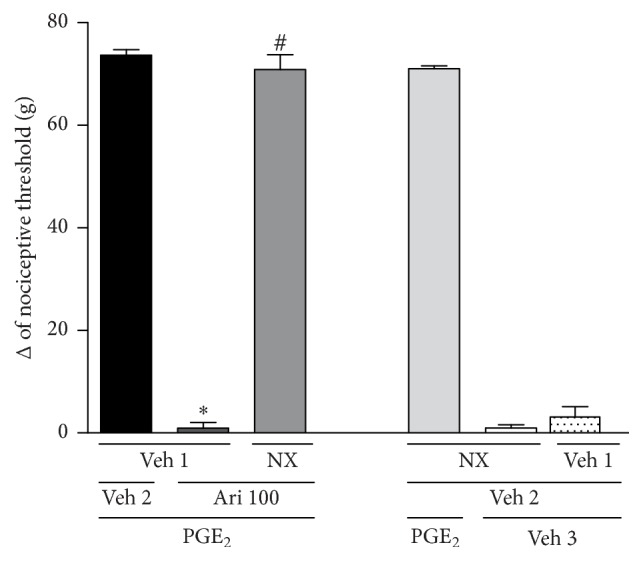
Naloxone antagonizes aripiprazole-induced antinociceptive effect against the hyperalgesic effect induced by PGE_2_ (PGE_2_, 2 *μ*g). Naloxone (NX; 50 *μ*g/paw) and aripiprazole (Ari; 100 *μ*g/paw) were given 140 min and 170 min after PGE_2_. The data are presented as mean and SEM (^*∗*^*p* < 0.05 compared with the PGE_2_ + Veh 1 + Veh 2; ^#^*p* < 0.05 compared with the PGE_2_ + Veh 1 + aripiprazole 100 *μ*g group; ANOVA followed by the Bonferroni test; *n* = 4 per group).

The injection of aripiprazole (100 *μ*g/paw) into the right hind paw produced an antinociceptive response against PGE_2_-induced hyperalgesia (2 *μ*g/paw, Figure 1). To verify the involvement of opioid system in this effect, the mice were pretreated with nonselective opioid receptor antagonist naloxone. Naloxone (50 *μ*g/paw) antagonized peripheral antinociceptive response of aripiprazole (100 *μ*g/paw) [*F*_(4,15)_ = 553.8; *p* < 0.0001]. When injected alone, naloxone did not induce antinociception or inhibit PGE_2_-induced hyperalgesia.

**Figure 2:**
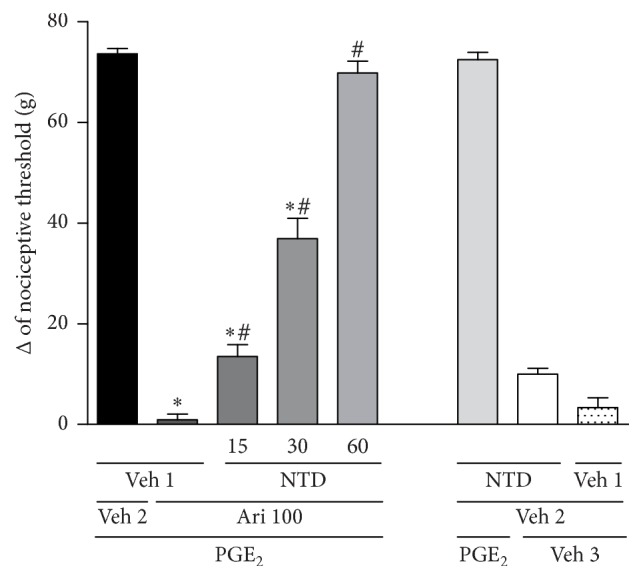
Naltrindole antagonizes the aripiprazole-induced antinociceptive effect against the hyperalgesic effect induced by PGE_2_ (PGE_2_, 2 *μ*g). Naltrindole (NTD; 15, 30, and 60 *μ*g/paw) and aripiprazole (Ari; 100 *μ*g/paw) were given 140 min and 170 min after PGE_2_. The data are presented as mean and SEM (^*∗*^*p* < 0.05 compared with the PGE_2_ + Veh 1 + Veh 2; ^#^*p* < 0.05 compared with the PGE_2_ + Veh 1 + aripiprazole 100 *μ*g group; ANOVA followed by the Bonferroni test; *n* = 4 per group).

**Figure 3:**
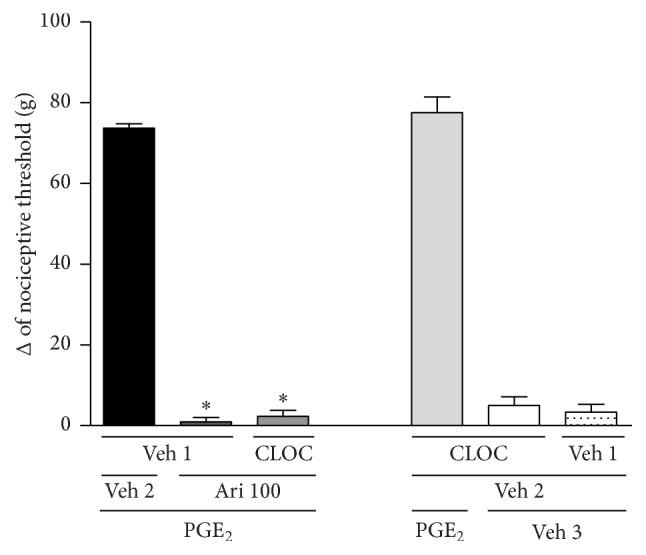
Clocinnamox did not antagonize the aripiprazole-induced antinociceptive effect against the hyperalgesic effect induced by PGE_2_ (PGE_2_, 2 *μ*g). Clocinnamox (CLOC; 40 *μ*g/paw) and aripiprazole (Ari; 100 *μ*g/paw) were given 140 min and 170 min after PGE_2_. The data are presented as mean and SEM (^*∗*^*p* < 0.05 compared with the PGE_2_ + Veh 1 + Veh 2; ANOVA followed by the Bonferroni test; *n* = 4 per group).

**Figure 4:**
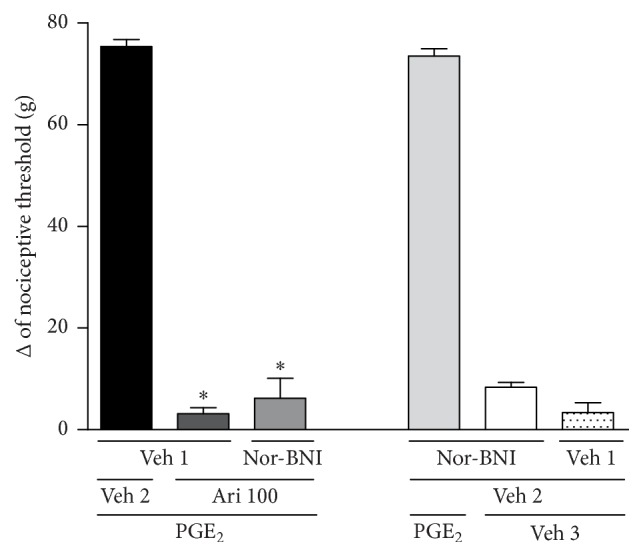
Nor-binaltorphimine did not antagonize the aripiprazole-induced antinociceptive effect against the hyperalgesic effect induced by PGE_2_ (PGE_2_, 2 *μ*g). Nor-binaltorphimine (nor-BNI; 200 *μ*g/paw) and aripiprazole (Ari; 100 *μ*g/paw) were given 140 min and 170 min after PGE_2_. The data are presented as mean and SEM (^*∗*^*p* < 0.05 compared with the PGE_2_ + Veh 1 + Veh 2; ANOVA followed by the Bonferroni test; *n* = 4 per group).

Once the involvement of opioid receptors in the mechanism of aripiprazole-induced antinociception was confirmed, the next step was to assess specifically which opioid receptor was involved in this process. The *μ*-, *κ*-, and *δ*-opioid receptor antagonists, clocinnamox (40 *μ*g/paw), nor-BNI (200 *μ*g/paw), or naltrindole (15, 30, and 60 *μ*g/paw), were injected prior to aripiprazole (100 *μ*g/paw, the dose required to reverse almost 100% of nociception). Naltrindole was able to inhibit the antinociceptive effect induced by aripiprazole in a dose-dependent manner [*F*_(6,21)_ = 247.4; *p* < 0.0001], Figure 2. However, neither clocinnamox [*F*_(4,15)_ = 397.1; *p* < 0.0001] nor nor-BNI [*F*_(4,15)_ = 380.9; *p* < 0.0001] blocked the antinociceptive response of aripiprazole, Figures 3 and 4. None of the compounds affected the nociceptive effect of PGE_2_ by themselves.

**Figure 5:**
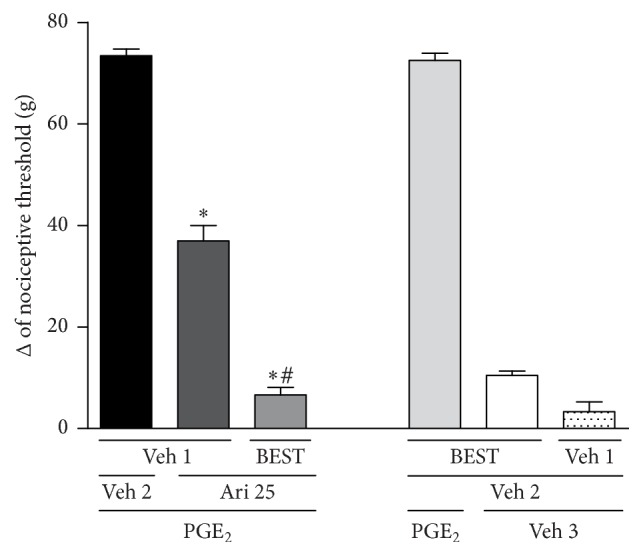
Bestatin potentiates the aripiprazole-induced antinociceptive effect against the hyperalgesic effect induced by PGE_2_ (PGE_2_, 2 *μ*g). Bestatin (BEST; 400 *μ*g/paw) and aripiprazole (Ari; 25 *μ*g/paw) were given 140 min and 170 min after PGE_2_. The data are presented as mean and SEM (^*∗*^*p* < 0.05 compared with the PGE_2_ + Veh 1 + Veh 2 group; ^#^*p* < 0.05 compared with the PGE_2_ + Veh 1 + aripiprazole 25 *μ*g group; ANOVA followed by Bonferroni test; *n* = 4 per group).

To evaluate the involvement of endogenous opioid peptides in the antinociceptive effect mediated by aripiprazole, the animals were treated with intraplantar injection of bestatin (400 *μ*g/paw). This administration increased the peripheral antinociceptive effect of aripiprazole (25 *μ*g/paw) [*F*_(4,15)_ = 331.5; *p* < 0.0001], the dose required to induce about 50% of antinociception, Figure 5. Bestatin alone did not affect the nociceptive effect of PGE_2_.

## 4. Discussion

This study evaluated the mechanisms of peripheral antinociception induced by aripiprazole, an antipsychotic drug that acts as a partial agonist at dopamine D_2_ receptor. The increased nociceptive response was induced by PGE_2_, which sensitizes primary afferent neurons and provokes hyperalgesia to a mechanical stimulus [18]. Previous work showed that aripiprazole prevented PGE_2_ effects in this model through activation of dopamine D_2_ and serotonin 5-HT_1A_ receptors [1]. However, considering the complex mechanisms modulating nociceptive processing, we do not rule out the possibility that additional mechanisms might contribute to the antinociceptive effect of aripiprazole, for example, the opioid system.

Opioids exert their effects through the Gi protein-coupled receptors *μ*, *δ*, and *κ* [19]. Their antinociceptive effects are well-established in different animal models, such as formalin [20–22] and tail flick [2, 23] tests.

In this work, naloxone, a nonselective opioid receptor antagonist, inhibited the peripheral antinociception induced by aripiprazole. The role of the *μ*-, *δ*-, and *κ*-opioid receptors was investigated using their selective antagonists clocinnamox, naltrindole, and nor-binaltorphimine, respectively. Our data indicated that only *δ*-opioid antagonist was able to reverse the peripheral antinociception induced by aripiprazole. This result is in agreement with several studies suggesting a role of *δ*-opioid receptor in peripheral antinociceptive effects [24–26]. Izquierdo and coworkers demonstrated that the peripheral administration of mangiferin produced a reduction of nociception in response to the formalin test, mediated by *δ*-receptors peripherally [26]. In addition, *δ*-receptors also mediated peripheral antinociception of the potent analgesic peptide, crotalphine, in a model of cancer pain induced by intraplantar injection of Walker 256 carcinoma cells [27]. In line with these data, the *δ*-opioid receptor agonist, SNC80, induced peripheral antinociceptive effect [28, 29]. Finally, PnPP-19, a spider toxin peptide, induces peripheral antinociception through *δ*-opioid receptor in rats [30]. Altogether, these results support our findings that aripiprazole induces peripheral antinociceptive effects through facilitation of the opioid system, particularly the *δ*-opioid receptor.

In the CNS, opioid receptors are expressed in subcortical regions of the brain (thalamus, cerebral cortex, periaqueductal grey, rostral ventromedial medulla, and amygdala, among others), from which descending pain-modulating pathways originate, and also in the dorsal horn of the spinal cord, an important area that sends nociceptive inputs to the brain and also a primary action site for opioids analgesic effects [9, 31–34]. In addition to this, at the peripheral level, the opioid receptors are expressed not only in neuronal cells [35, 36], but also in immune cells (macrophages and neutrophils) as well as keratinocytes [37]. Similarly, D_2_ receptors are also expressed at significant levels in the nucleus accumbens, ventral tegmental area, hypothalamus, cortical areas, septum, amygdala, and hippocampus [38–41]. Moreover, D_2_ and 5-TH_1A_ receptors are also found in dorsal root ganglia (DRG) and keratinocytes, showing the peripheral presence of these receptors [42–45].

It remains unclear, however, how aripiprazole facilitates the endogenous opioid system. D_2_ receptor might interact with the opioid system at a downstream level, such as by facilitating *δ*-opioid and D_2_ receptor heterodimerization or by interfering with signal transduction processes. Neurochemical works have shown that dopamine and opioid systems are one of the major endogenous systems involved in several behaviors, such as pain perception and its modulatory mechanisms, the reward system, dependence, and fear control [1, 2, 46–48]. Furthermore, previous studies showed that the systemic administration of *δ*-opioid receptor agonists facilitates dopaminergic activity in the striatum and forebrain regions, as determined by high-affinity dopamine uptake and turnover rates [49, 50]. Le Moine and coworkers demonstrated that the major striatopallidal neurons express D_2_ receptors and enkephalin [51], suggesting an interaction between the dopamine and opioid systems. It remains to be investigated if these mechanisms also operate to modulate pain responses in the periphery.

Another possibility is that D_2_ receptor partial agonist facilitates the release of endogenous opioids which, in turn, activate the *δ*-opioid receptor. The result showing that bestatin, an aminopeptidase-N inhibitor, potentiated the peripheral antinociceptive effect induced by a low dose of aripiprazole supports this possibility. D_2_ receptor could interact with the beta-gamma complex (G*βγ*) signaling and activate phospholipase C (PLC). This would lead to IP_3_ receptor activation, resulting in increase in intracellular calcium [52, 53]. Calcium increase would stimulate the synthesis of proenkephalin (PENK) which, in turn, activates the *δ*-opioid receptor to reduce nociceptive response.

In conclusion, our data suggest that the peripheral antinociceptive effect of aripiprazole is associated with facilitation of endogenous opioid activity through the *δ*-opioid receptors. The therapeutic potential of aripiprazole for the treatment of certain types of pain warrants further investigation.

## References

[1] Almeida-Santos A. F., Ferreira R. C. M., Duarte I. D., Aguiar D. C., Romero T. R. L., Moreira F. A. (2015). The antipsychotic aripiprazole induces antinociceptive effects: Possible role of peripheral dopamine D2 and serotonin 5-HT1A receptors. *European Journal of Pharmacology*.

[2] Almeida-Santos A. F., Gobira P. H., Souza D. P. (2014). The antipsychotic aripiprazole selectively prevents the stimulant and rewarding effects of morphine in mice. *European Journal of Pharmacology*.

[3] Aira Z., Barrenetxea T., Buesa I., del Caño G. G., Azkue J. J. (2016). Dopamine D1-like receptors regulate constitutive, *μ*-opioid receptor-mediated repression of use-dependent synaptic plasticity in dorsal horn neurons: More harm than good?. *Journal of Neuroscience*.

[4] Akil H., Owens C., Gutstein H., Taylor L., Curran E., Watson S. (1998). Endogenous opioids: Overview and current issues. *Drug and Alcohol Dependence*.

[5] Jordan B., Devi L. A. (1998). Molecular mechanisms of opioid receptor signal transduction. *British Journal of Anaesthesia*.

[6] Burris K. D., Molski T. F., Xu C. (2002). Aripiprazole, a novel antipsychotic, is a high-affinity partial agonist at human dopamine D2 receptors. *Journal of Pharmacology and Experimental Therapeutics*.

[7] Stark A. D., Jordan S., Allers K. A. (2007). Interaction of the novel antipsychotic aripiprazole with 5-HT1A and 5-HT2A receptors: Functional receptor-binding and in vivo electrophysiological studies. *Psychopharmacology*.

[8] Shapiro D. A., Renock S., Arrington E. (2003). Aripiprazole, a novel atypical antipsychotic drug with a unique and robust pharmacology. *Neuropsychopharmacology*.

[9] Kieffer B. L., Evans C. J. (2009). Opioid receptors: From binding sites to visible molecules in vivo. *Neuropharmacology*.

[10] França D. S., Ferreira-Alves D. L., Duarte I. D. G. (2006). Endogenous opioids mediate the hypoalgesia induced by selective inhibitors of cyclo-oxygenase 2 in rat paws treated with carrageenan. *Neuropharmacology*.

[11] Nakamura M., Ferreira S. H. (1988). Peripheral analgesic action of clonidine: mediation by release of endogenous enkephalin-like substances. *European Journal of Pharmacology*.

[12] Nakamura M., Lico M. C. (1988). Peripheral modulation of pain in conscious guinea pigs: effect of morphine, nalorphine and clonidine. *Brazilian Journal of Medical and Biological Research*.

[13] Lima Romero T. R., de Castro Perez A., de Francischi J. N., Gama Duarte I. D. (2009). Probable involvement of *α*2C-adrenoceptor subtype and endogenous opioid peptides in the peripheral antinociceptive effect induced by xylazine. *European Journal of Pharmacology*.

[14] Randall L. O., Selitto J. J. (1957). A method for measurement of analgesic activity on inflamed tissue. *Archives internationales de pharmacodynamie et de therapie*.

[15] Kawabata A., Nishimura Y., Takagi H. (1992). l‐Leucyl‐l‐arginine, naltrindole and d‐arginine block antinociception elicited by l‐arginine in mice with carrageenin‐induced hyperalgesia. *British Journal of Pharmacology*.

[16] Romero T. R. L., Pacheco D. D. F., Duarte I. D. G. (2013). Xylazine induced central antinociception mediated by endogenous opioids and *μ*-opioid receptor, but not *δ*-or *κ*-opioid receptors. *Brain Research*.

[17] Pacheco C. M. F., Queiroz-Junior C. M., Maltos K. L. M. (2008). Crucial role of peripheral *κ*-opioid receptors in a model of periodontal disease in rats. *Journal of Periodontal Research*.

[18] Sachs D., Villarreal C., Cunha F., Parada C., Ferreira S. (2009). The role of PKA and PKC*η* pathways in prostaglandin E2-mediated hypernociception. *British Journal of Pharmacology*.

[19] Feng Y., He X., Yang Y., Chao D., Lazarus L. H., Xia Y. (2012). Current research on opioid receptor function. *Current Drug Targets*.

[20] Dubuisson D., Dennis S. G. (1978). The formalin test: a quantitative study of the analgesic effects of morphine, meperidine, and brain stem stimulation in rats and cats. *Pain*.

[21] Hunskaar S., Fasmer O. B., Hole K. (1985). Formalin test in mice, a useful technique for evaluating mild analgesics. *Journal of Neuroscience Methods*.

[22] Sandkühler J. (2009). Models and mechanisms of hyperalgesia and allodynia. *Physiological Reviews*.

[23] Cecchi M., Capriles N., Watson S. J., Akil H. (2008). Differential responses to morphine-induced analgesia in the tail-flick test. *Behavioural Brain Research*.

[24] Veloso C. D. C., Rodrigues V. G., Ferreira R. C. M. (2014). Tingenone, a pentacyclic triterpene, induces peripheral antinociception due to opioidergic activation. *Planta Medica*.

[25] Da Fonseca Pacheco D., Klein A., De Castro Perez A., Da Fonseca Pacheco C. M., De Francischi J. N., Duarte I. D. G. (2008). The *μ*-opioid receptor agonist morphine, but not agonists at *δ*- Or *κ*-opioid receptors, induces peripheral antinociception mediated by cannabinoid receptors. *British Journal of Pharmacology*.

[26] Izquierdo T., Espinosa De Los Monteros-Zuñiga A., Cervantes-Durán C., Lozada M. C., Godínez-Chaparro B. (2013). Mechanisms underlying the antinociceptive effect of mangiferin in the formalin test. *European Journal of Pharmacology*.

[27] Brigatte P., Konno K., Gutierrez V. P. (2013). Peripheral kappa and delta opioid receptors are involved in the antinociceptive effect of crotalphine in a rat model of cancer pain. *Pharmacology Biochemistry and Behavior*.

[28] Romero T. R. L., Guzzo L. S., Duarte I. D. G. (2012). Mu, Delta, and Kappa opioid receptor agonists induce peripheral antinociception by activation of endogenous noradrenergic system. *Journal of Neuroscience Research*.

[29] Pacheco D. D. F., Pacheco C. M. D. F., Duarte I. D. G. (2012). Peripheral antinociception induced by *δ*-opioid receptors activation, but not *μ*- or *κ*-, is mediated by Ca^2+^-activated Cl^−^ channels. *European Journal of Pharmacology*.

[30] Freitas A. C. N., Pacheco D. F., MacHado M. F. M., Carmona A. K., Duarte I. D. G., De Lima M. E. (2016). PnPP-19, a spider toxin peptide, induces peripheral antinociception through opioid and cannabinoid receptors and inhibition of neutral endopeptidase. *British Journal of Pharmacology*.

[31] Stein C., Lang L. J. (2009). Peripheral mechanisms of opioid analgesia. *Current Opinion in Pharmacology*.

[32] Stein C., Schäfer M., Machelska H. (2003). Attacking pain at its source: New perspectives on opioids. *Nature Medicine*.

[33] Rau K. K., Caudle R. M., Cooper B. Y., Johnson R. D. (2005). Diverse immunocytochemical expression of opioid receptors in electrophysiologically defined cells of rat dorsal root ganglia. *Journal of Chemical Neuroanatomy*.

[34] Gendron L., Lucido A. L., Mennicken F. (2006). Morphine and pain-related stimuli enhance cell surface availability of somatic *δ*-opioid receptors in rat dorsal root ganglia. *Journal of Neuroscience*.

[35] Coggeshall R. E., Zhou S., Carlton S. M. (1997). Opioid receptors on peripheral sensory axons. *Brain Research*.

[36] Stein C., Hassan A. H., Przewlocki R., Gramsch C., Peter K., Herz A. (1990). Opioids from immunocytes interact with receptors on sensory nerves to inhibit nociception in inflammation.. *Proceedings of the National Academy of Sciences*.

[37] Bigliardi P. L., Tobin D. J., Gaveriaux-Ruff C., Bigliardi-Qi M. (2009). Opioids and the skin - Where do we stand?. *Experimental Dermatology*.

[38] Missale C., Nash S. R., Robinson S. W., Jaber M., Caron M. G. (1998). Dopamine receptors: from structure to function. *Physiological Reviews*.

[39] Gerfen C. R. (2000). Molecular effects of dopamine on striatal-projection pathways. *Trends in Neurosciences*.

[40] Clifford J. J., Usiello A., Vallone D., Kinsella A., Borrelli E., Waddington J. L. (2000). Topographical evaluation of behavioural phenotype in a line of mice with targeted gene deletion of the D2 dopamine receptor. *Neuropharmacology*.

[41] Seeman P. (2006). Targeting the dopamine D2 receptor in schizophrenia. *Expert Opinion on Therapeutic Targets*.

[42] Xie G.-X., Jones K., Peroutka S. J., Palmer P. P. (1998). Detection of mRNAs and alternatively spliced transcripts of dopamine receptors in rat peripheral sensory and sympathetic ganglia. *Brain Research*.

[43] Wu S.-X., Zhu M., Wang W., Wang Y.-Y., Li Y.-Q., Yew D. T. (2001). Changes of the expression of 5-HT receptor subtype mRNAs in rat dorsal root ganglion by complete Freund's adjuvant-induced inflammation. *Neuroscience Letters*.

[44] Fuziwara S., Suzuki A., Inoue K., Denda M. (2005). Dopamine D2-like receptor agonists accelerate barrier repair and inhibit the epidermal hyperplasia induced by barrier disruption. *Journal of Investigative Dermatology*.

[45] Nordlind K., Azmitia E. C., Slominski A. (2008). The skin as a mirror of the soul: Exploring the possible roles of serotonin. *Experimental Dermatology*.

[46] Roberts D. C. S., Koob G. F. (1982). Disruption of cocaine self-administration following 6-hydroxydopamine lesions of the ventral tegmental area in rats. *Pharmacology, Biochemistry and Behavior*.

[47] Fink J. S., Smith G. P. (1980). Mesolimbic and mesocortical dopaminergic neurons are necessary for normal exploratory behavior in rats. *Neuroscience Letters*.

[48] Lesniak A., Lipkowski A. W. (2011). Opioid peptides in peripheral pain control. *Acta Neurobiological Experimentals*.

[49] Spanagel R., Herz A., Bals-Kubik R., Shippenberg T. S. (1991). *β*-Endorphin-induced locomotor stimulation and reinforcement are associated with an increase in dopamine release in the nucleus accumbens. *Psychopharmacology*.

[50] Spanagel R., Herz A., Shippenberg T. S. (1991). Modulation of the mesolimbic dopaminergic system by *β*-endorphin-(1-27) as assessed by microdialysis. *European Journal of Pharmacology*.

[51] Le Moine C., Normand E., Guitteny A. F., Fouque B., Teoule R., Bloch B. (1990). Dopamine receptor gene expression by enkephalin neurons in rat forebrain. *Proceedings of the National Academy of Sciences of the United States of America*.

[52] Hernádez-López S., Tkatch T., Perez-Garci E. (2000). D2 dopamine receptors in striatal medium spiny neurons reduce L-type Ca^2+^ currents and excitability via a novel PLC[beta]1-IP3-calcineurin-signaling cascade. *Journal of Neuroscience*.

[53] Hahm S. H., Chen Y., Vinson C., Eiden L. E. (2003). A Calcium-Initiated Signaling Pathway Propagated through Calcineurin and cAMP Response Element-Binding Protein Activates Proenkephalin Gene Transcription after Depolarization. *Molecular Pharmacology*.

